# Region-Wise Bézier Intensity Augmentation for Domain-Generalized Brain Tumor Segmentation with a Mamba U-Net

**DOI:** 10.3390/jcm15145508

**Published:** 2026-07-14

**Authors:** Mustafa Yurdakul, Merve Ersoy, Faruk Özger, Ishak Pacal

**Affiliations:** 1 Department of Computer Engineering, Faculty of Engineering and Natural Sciences, Kırıkkale University, Kırıkkale 71450, Türkiye; mustafayurdakul@kku.edu.tr; 2 Department of Software Engineering, Faculty of Engineering and Natural Sciences, Istanbul Topkapı University, Istanbul 34087, Türkiye; merveersoy@topkapi.edu.tr; 3 Department of Computer Engineering, Faculty of Engineering, Iğdır University, Iğdır 76000, Türkiye; ishak.pacal@igdir.edu.tr; 4 Department of Electronics and Information Technologies, Faculty of Architecture and Engineering, Nakhchivan State University, Nakhchivan AZ 7012, Azerbaijan; 5 Department of Computer Engineering, Faculty of Engineering and Natural Sciences, Fenerbahce University, Istanbul 34758, Türkiye

**Keywords:** brain tumor segmentation, MRI, Mamba, Swin-UMamba, data augmentation, Bézier curve, domain generalization, deep learning

## Abstract

**Background/Objectives:** Robust brain-tumor segmentation on contrast-enhanced MRI remains limited by scanner-dependent intensity shifts, scarce annotations, and evaluation protocols that may leak patient-specific information. We propose BA-SwinMamba, a region-wise Bézier intensity augmentation framework built on Swin-UMamba, a selective state-space U-Net that combines hierarchical Swin-style visual modeling with Mamba’s linear-complexity long-range sequence representation. **Materials and Methods:** During training, independent monotonic or non-monotonic Bézier transfer functions are sampled for tumor and background regions, perturbing lesion-to-background contrast while preserving the binary mask geometry. Fourteen convolutional, transformer-based, and state-space segmentation models were evaluated on the Cheng brain-tumor dataset, comprising 3064 contrast-enhanced T1-weighted slices from 233 patients, using a strictly patient-level five-fold protocol. Single-source domain generalization was assessed by training only on Cheng and testing, without fine-tuning, on two independent target datasets. **Results:** BA-SwinMamba achieved 89.6% Dice, 82.0% IoU, and 5.9-pixel HD95 on the source domain, outperforming the plain Swin-UMamba backbone by 1.7 Dice points. The benefit was larger under domain shift: mean target-domain Dice increased from 72.7% with Swin-UMamba to 78.3% with BA-SwinMamba. Ablation analysis showed that replacing global Bézier augmentation with the proposed region-wise formulation added 1.5 Dice points. **Conclusions:** The method introduces no inference-time cost because augmentation is disabled after training, without modifying the deployed network or requiring target-domain labels during model optimization or tuning. The results indicate that lesion-aware intensity perturbation can improve cross-dataset robustness of Mamba-based 2D brain-tumor segmentation, while wider volumetric and multi-institutional validation remains necessary.

## 1. Introduction

Brain and other central nervous system tumors are among the most serious oncological diseases. Although they are less common than breast, lung, or prostate tumors, they are associated with disproportionate morbidity and mortality because they develop within a confined and functionally critical organ [1]. Tumors are broadly classified as primary lesions, which originate in brain tissue or its coverings, and metastatic lesions, which spread from other parts of the body. Three primary tumor types dominate adult neuro-oncological imaging and define the dataset used in this study. Meningiomas arise from the meninges that surround the brain and are usually slow-growing and extra-axial. Gliomas originate from glial cells, range in malignancy from low-grade tumors to highly aggressive glioblastomas, and are typically intra-axial with infiltrative and irregular margins. Pituitary tumors develop in the pituitary gland at the skull base and, although often benign, may disrupt hormonal balance and compress neighboring structures. The precise causes of most brain tumors remain unknown. Established risk factors are limited and include prior therapeutic ionizing radiation and a small number of hereditary syndromes, whereas the vast majority of cases are sporadic [2].

Magnetic resonance imaging is the modality of choice for detecting and characterizing these tumors because it provides detailed soft-tissue contrast without ionizing radiation and offers several complementary imaging contrasts. T1-weighted sequences acquired after administration of a gadolinium-based contrast agent are particularly informative, as areas with disruption of the blood–brain barrier enhance brightly and reveal the active tumor and its boundary [2]. In current clinical practice, radiologists inspect these images and, when quantitative analysis is required, manually delineate the tumor slice by slice. Histopathological examination of tissue obtained through biopsy or resection remains the diagnostic gold standard; however, imaging guides initial detection, surgical and radiotherapy planning, and longitudinal follow-up.

Manual delineation, however, is time-consuming, tedious, and difficult to scale to the volume of imaging generated in routine clinical care. It is also affected by substantial inter- and intra-observer variability: two experts, or the same expert at different times, may draw noticeably different boundaries, particularly in regions where the tumor gradually blends into the surrounding tissue. Classical automated approaches based on intensity thresholding, region growing, or atlas registration can reduce the manual burden, but they are brittle and depend on hand-tuned assumptions that often fail under the intensity inhomogeneity, partial-volume effects, and appearance variability typical of MRI. These limitations have motivated the shift toward learning-based segmentation.

Deep convolutional networks, particularly the U-Net encoder–decoder architecture, have become the standard approach for medical image segmentation. In U-Net, the contracting encoder captures contextual information, while the expanding decoder, supported by skip connections, restores spatial detail to produce accurate boundaries [3]. Building on this architecture, self-configuring pipelines such as nnU-Net have emerged as strong baselines [4]. Attention-gated variants further refined boundary delineation [5]. Since then, two structural constraints have shaped the field. Convolutional networks capture long-range relationships only indirectly through stacked local filters, whereas vision transformers can model global context directly but at a computational cost that grows quadratically with image size. Transformer-based architectures have therefore been adapted for medical segmentation in models such as TransUNet [6]. Swin UNETR introduced a hierarchical shifted-window encoder for this purpose [7]. Selective state-space models, introduced through Mamba [8], offer a third direction by modeling long sequences in linear time while still propagating information across the entire image. Their medical adaptations, including U-Mamba and Swin-UMamba, have matched or outperformed convolutional and transformer baselines, particularly when the Mamba encoder is pretrained on ImageNet to mitigate the scarcity of labelled medical data [9].

Stronger backbones do not by themselves address two methodological weaknesses that remain common in 2D brain-tumor segmentation. First, annotated MRI data are limited and visually heterogeneous, whereas standard geometric augmentations mainly perturb spatial configuration rather than scanner- or protocol-dependent appearance. Intensity remapping with Bézier curves offers a controlled way to vary image contrast without changing the tumor mask and has been used as an appearance-level augmentation strategy in medical image analysis [10]. Second, several studies on the Cheng dataset have used random slice-level partitioning, which can place correlated slices from the same patient in both training and test partitions. Such leakage can inflate Dice and IoU values and make reported results difficult to compare. The present study therefore combines region-wise Bézier intensity augmentation with Swin-UMamba and evaluates the model under patient-level five-fold cross-validation and external cross-dataset testing.

## 2. Related Works

Research on two-dimensional brain-tumor segmentation has largely converged on the Cheng dataset, from which a clear performance hierarchy has emerged. Comparative studies have benchmarked several U-Net variants on this dataset, including U-Net, attention U-Net, deep residual U-Net, ResUNet++, and recurrent-residual U-Net, with the recurrent-residual variant reported as the most consistent within this family [11]. Subsequent designs incorporated attention mechanisms and stronger encoders. A multi-scale attention U-Net with an EfficientNet-B4 encoder reported a Dice score of 0.93 and an IoU of 0.8795 [12]. A lightweight spatial-attention U-Net achieved a Dice score close to 0.93 with substantially fewer parameters [13]. A two-headed UNet-EfficientNet performing segmentation and classification jointly reported a post-processed Dice score of 0.94, which increased to 0.96 when used in an ensemble [14]. In parallel, state-space models entered medical segmentation through Swin-UMamba [9]. U-Mamba applied this design directly to biomedical image segmentation [15]. For volumetric data, SegMamba extended state-space modeling to three dimensions [16]. However, Mamba-based brain-tumor segmentation studies have so far focused mainly on the multimodal three-dimensional BraTS benchmark rather than the two-dimensional contrast-enhanced setting [17]. Compute-efficient diagnostic architectures based on directional split convolutions have also demonstrated competitive performance in this setting [18]. Beyond segmentation, the Cheng dataset is also widely used for tumor classification, where attention-augmented CNNs, such as an explainable EfficientNetV2 with an MLP-Mixer attention head, achieve high accuracy [19]. Comparative evaluations of lightweight CNN and vision-transformer models report competitive multi-class brain-tumor accuracy [20]. Lightweight vision-transformer designs have shown comparable gains for medical diagnosis [21]. Systematic surveys of cutting-edge deep learning models further consolidate best practices for brain tumor classification [22]. Table 1 summarizes representative segmentation studies on the Cheng dataset.

Beyond data augmentation, two additional families of domain-generalization methods are worth noting. Feature-alignment approaches aim to learn domain-invariant representations, for example through adversarial training that makes features indistinguishable across domains [23]. Test-time adaptation, by contrast, adapts a trained model to each incoming distribution by minimizing the entropy of its predictions on unlabelled target data [24]. Both approaches are complementary to the input-space strategy adopted in this study, and neither requires access to the augmentation pipeline. Within the augmentation family, the proposed transform can be positioned against simpler intensity operations. Gamma correction applies a single global power-law transformation, while histogram matching imposes a global monotonic mapping onto a reference distribution. As both operate uniformly over the entire image, they cannot decouple the lesion from its surrounding background. The Bézier-curve formulation instead defines a smooth and potentially non-monotonic family of intensity remappings [10]. Style-augmentation methods have likewise improved cross-modality generalization [25]. Rethinking augmentation for single-source domain generalization further motivates such perturbations [26]. In its region-wise form, it transforms the tumor and background using independent curves, thereby explicitly diversifying the lesion-to-background contrast that determines the segmentation boundary.

Two limitations in this literature define the scope of the present work. First, high Dice scores reported on the Cheng dataset often come from random slice-level splits, so their relationship to patient-level performance remains uncertain. Second, cross-dataset testing is still uncommon, despite the fact that MRI intensity distributions vary across scanners, protocols, preprocessing pipelines, and institutions. Bézier-based intensity augmentation has shown value in domain-generalization settings, but it has mainly been applied as a global image-level transform rather than as a region-wise perturbation of lesion and background contrast [10]. Style-augmentation methods have likewise applied appearance changes at the global image level [25]. Work rethinking single-source augmentation has used similar global intensity perturbations [26]. This study makes three contributions. First, it introduces a region-wise Bézier intensity augmentation that independently remaps tumor and background intensities while preserving mask geometry. Second, it benchmarks fourteen convolutional, transformer-based, and state-space segmentation models under a patient-level five-fold protocol. Third, it evaluates single-source domain generalization on two unseen datasets using ablation experiments, class-stratified Dice, cross-domain Dice, qualitative segmentation examples, and pixel-level error analysis.

## 3. Materials and Methods

### 3.1. Dataset and Preprocessing

**Figure 1:**
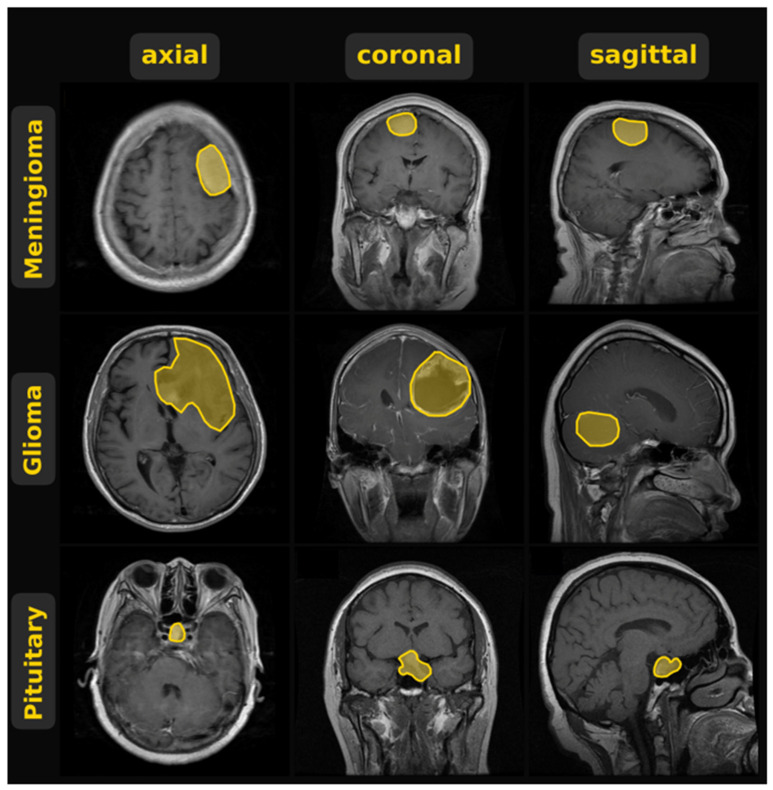
Example Cheng slices arranged by tumor type (rows) and imaging plane (columns): each row corresponds to one tumor type (meningioma, glioma, and pituitary tumor), and each column corresponds to one imaging plane (axial, coronal, and sagittal). The tumor region is highlighted in yellow.

The source dataset was the publicly available Cheng brain-tumor MRI dataset, which contains 3064 contrast-enhanced T1-weighted slices from 233 patients [27]. The dataset includes meningioma, glioma, and pituitary tumor cases, with 708, 1426, and 930 slices, respectively, acquired in axial, coronal, and sagittal planes. Each sample provides an MR slice, a radiologist-delineated binary tumor mask, a tumor-type label, and a patient identifier. Figure 1 shows representative examples across tumor types and imaging planes, with the annotated tumor boundary overlaid to illustrate the segmentation target.

**Figure 2:**
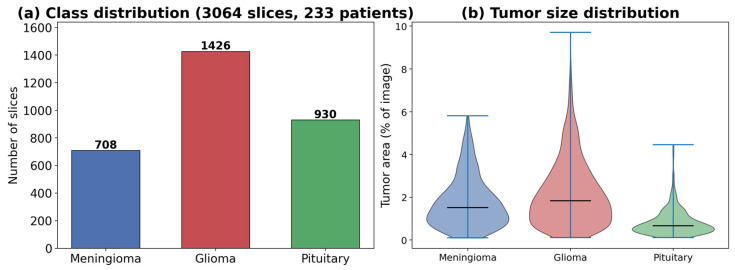
Dataset composition: (**a**) slices per type; (**b**) tumor-area distribution per type.

The dataset is class-imbalanced, and the tumor types differ substantially in size. As shown in Figure 2, pituitary tumors occupy a smaller fraction of the slice, with a median area of 0.67%, compared with meningioma (1.52%) and glioma (1.84%). This makes the pituitary class the most challenging and motivates the reporting of class-stratified scores.

The unit of separation was the patient rather than the slice. Because each patient may contribute multiple correlated slices, random slice-level splitting can place anatomically related samples from the same patient in both training and test partitions. We therefore used stratified group five-fold cross-validation at the patient identifier level. All slices from a given patient were assigned to a single fold, while tumor-type proportions were preserved as closely as possible. Source-domain performance was reported as the mean across the five held-out patient-level folds. 

Two external datasets were used only for cross-domain testing and were not used for training, early stopping, hyperparameter selection, or model selection. The first target set was the de-duplicated BRISC dataset, a contrast-enhanced T1 collection with expert tumor masks [28]. Cases overlapping with the Cheng dataset were removed before evaluation. The second target set was derived from T1c images in a BraTS release and represents a different acquisition and preprocessing regime, including skull stripping, co-registration, and resampling. Its tumor subregion labels were merged into a single foreground class to match the binary segmentation target. All source and target images were min–max normalized to [0, 1], resized to 256 × 256 pixels, and replicated to three channels for compatibility with the ImageNet-pretrained encoder. Masks were resized with nearest-neighbor interpolation. This protocol keeps preprocessing consistent across datasets, although the external results should still be interpreted as cross-dataset robustness rather than as a full estimate of clinical generalization.

### 3.2. Region-Wise Bézier Intensity Augmentation

Geometric augmentations such as flips and small rotations alter spatial configuration, but they do not directly model intensity and contrast variation across MRI acquisitions. To perturb appearance while preserving the annotation geometry, we use Bézier-based intensity remapping during training. Bézier curves provide a smooth, low-parameter non-linear mapping of normalized pixel intensities and have previously been used to represent object shapes for segmentation [29]. They have also been applied to interpretable structural modeling of MR images [30]. A cubic Bézier curve is defined by four control points $P_{0},P_{1},P_{2},P_{3}$ and a parameter t∈[0,1], as given in Equation (1),

(1) B(t)=∑i=03(3i)(1−t)3−it iPi.

**Figure 3:**
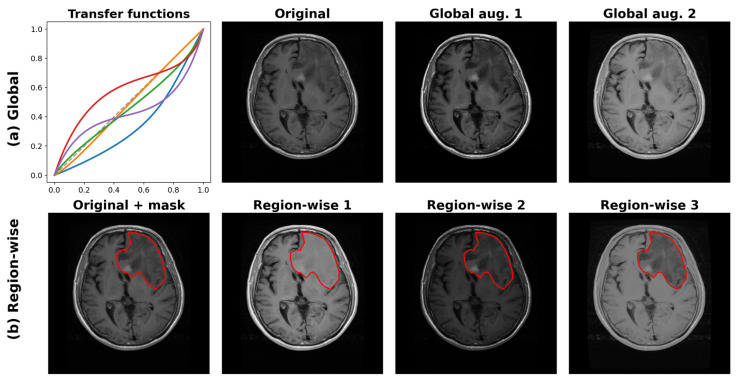
Bézier intensity augmentation: (**a**) global and (**b**) region-wise variants; the mask (red) is preserved. In the transfer-function panel (**top left** of (**a**)), the horizontal axis represents the input intensity and the vertical axis represents the remapped output, both normalized to [0, 1]. The dashed gray diagonal indicates the identity mapping, and each colored curve corresponds to a sampled cubic Bézier transfer function. Monotonic curves behave like smooth contrast or gamma adjustments, whereas non-monotonic curves invert the local intensity order to produce a stronger perturbation. In (**a**), the remaining columns present an original slice followed by two globally augmented samples. In (**b**), the columns show the original slice with the tumor mask outlined in red, followed by three region-wise samples in which the tumor and background are remapped using independent curves while the mask itself remains unchanged.

The endpoints in Equation (1) are fixed at $P_{0}=(0,0)$ and $P_{3}=(1,1)$. The two interior control points define an intensity transfer function f:[0,1]→[0,1], which remaps each normalized input intensity *I* to *f*(*I*). In the monotonic regime, the sampled control points are constrained so that the resulting mapping preserves intensity order and behaves as a smooth contrast transformation. In the non-monotonic regime, this ordering constraint is relaxed to generate stronger appearance perturbations. During training, the monotonic and non-monotonic regimes are sampled with probabilities $p_{mono}$ and 1 − $p_{mono}$, respectively. A tumor slice can be decomposed into foreground tumor and surrounding background, and the contrast between these two regions is central to boundary localization. We therefore apply the intensity transform in a region-wise manner. Two independent transfer functions, $f_{tum}$ and $f_{bg}$, are sampled and composed using the binary mask M, as defined in Equation (2).

(2) $$I^{\prime }\left(u\right)=M\left(u\right)\;f_{\mathrm{tum}}\left(I\left(u\right)\right)+\left(1-M\left(u\right)\right)\;f_{\mathrm{bg}}\left(I\left(u\right)\right),$$

for every pixel *u*. A narrow boundary band can be smoothed to avoid a visible seam between the two remapped regions. Equation (2) perturbs lesion-to-background contrast as a proxy for acquisition-dependent appearance variation, while the spatial mask is not modified. Figure 3 shows the global and region-wise variants on a representative slice. During training, the transform is applied on the fly with probability paug and combined with standard flips and small rotations applied jointly to the image and mask.

Each Bézier transfer function is represented as a cubic curve with endpoints fixed at (0, 0) and (1, 1). The two interior control points are sampled within a bounded range around the identity diagonal, so the strength of the perturbation is limited by a predefined deviation bound. When the sampled ordinates are ordered, the mapping remains monotonic and produces a smooth contrast-like transformation. When the ordering constraint is removed, the mapping can become non-monotonic and produces stronger intensity perturbations. The purpose of the non-monotonic regime is not to generate photorealistic MR images, but to expose the model to a wider range of lesion-background contrast configurations during training. This form of domain randomization follows prior reports in which intensity perturbation improved single-source generalization [10]. Style-augmentation methods have shown similar cross-domain benefits [25]. Rethinking single-source augmentation provides further support for this strategy [26]. Since only image intensities are changed and the binary mask is preserved, the spatial label remains valid for the augmented sample.

The tumor mask is used only to define the foreground and background regions for training-time intensity remapping. It is not concatenated with the image, not passed to the network as an input, and not used during inference. This also applies to cross-domain testing, where target masks are used only for evaluation after prediction. The procedure is therefore a label-aware augmentation strategy rather than a test-time mask-conditioned method. A formal radiologist assessment of the augmented images was not performed, and the visual plausibility of non-monotonic samples remains a limitation of the present study.

### 3.3. Swin-UMamba Segmentation Network

The segmentation backbone is Swin-UMamba, a Mamba-based U-Net variant [9]. It follows the encoder–decoder structure of U-Net [3] but uses selective state-space blocks in the feature extraction pathway instead of relying only on convolutional or attention-based operations.

Each selective state-space block is based on an SSM that maps an input signal *x*(*t*) to an output *y*(*t*) through a latent state *h*(t), as defined in Equation (3),

(3) $$h^{\prime }\left(t\right)=A\;h\left(t\right)+B\;x\left(t\right),\;\;y\left(t\right)=C\;h\left(t\right)+D\;x\left(t\right),$$

where $A\in R^{N\times N}$ is the state-transition matrix and B,C,D are learnable projections. To process discrete feature maps, the system is discretized with a zero-order hold of time step Δ, yielding the recurrence of Equation (4),

(4) $$\bar{A}=exp\left(\Delta A\right),\;\bar{B}={\left(\Delta A\right)}^{-1}\left(exp\left(\Delta A\right)-I\right)\;\Delta B,\;h_{k}=\bar{A}\;h_{k-1}+\bar{B}\;x_{k},\;y_{k}=C\;h_{k}.$$

In the selective (Mamba) variant, the parameters Δ,B,C are made functions of the input, so the block can adaptively retain or discard information along the sequence; this input dependence gives the SSM its content-aware, long-range behavior at a linear cost. Because an image has no single causal ordering, the visual state-space block unrolls the feature map along four directions and averages the per-direction outputs, as in Equation (5),

(5) $$\bar{y}\left(u\right)=1/4\sum_{d=1}^{4}{SSM}_{d}\left({scan}_{d}\left(x\right)\right)\left(u\right),$$

so that each spatial location u aggregates context from the entire slice while overall complexity stays linear in the number of pixels.

**Figure 4:**
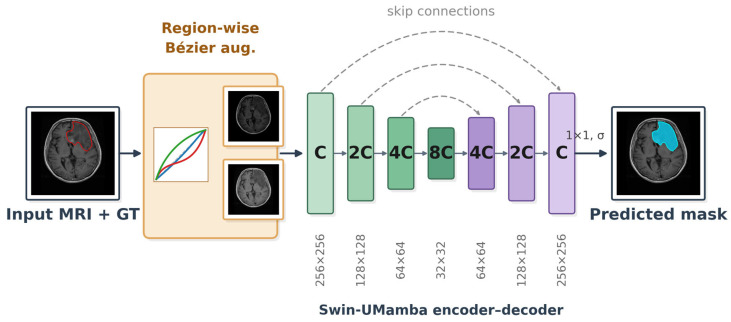
Overview of the BA-SwinMamba pipeline: region-wise Bézier augmentation feeding the Swin-UMamba encoder–decoder. Arrows indicate the direction of data flow through the network, and the shaded region highlights the region-wise Bézier intensity augmentation that is applied only during training.

The encoder follows a four-stage VMamba-Tiny layout [31]. This enables transfer from ImageNet-pretrained weights and reduces dependence on the limited number of annotated medical images [10]. A convolutional stem first embeds the input slice into patch tokens. The subsequent stages progressively reduce spatial resolution and increase channel width from C to 2C, 4C, and 8C. Each stage combines the four-direction scan in Equation (5) with depth-wise convolution, layer normalization, and a gated feed-forward module. Skip connections link encoder and decoder features at matching resolutions. The decoder upsamples the feature maps to the original resolution, and a final 1 × 1 convolution followed by a sigmoid activation generates the tumor probability map. Two decoder variants are evaluated: the standard convolutional decoder and the lighter Mamba decoder, denoted Swin-UMamba† in the original work. Figure 4 summarizes the training pipeline, including region-wise Bézier augmentation and the Swin-UMamba encoder-decoder.

### 3.4. Experimental Setup

All experiments were implemented in PyTorch 2.1 and trained on a single NVIDIA RTX 3090 GPU (NVIDIA Corporation, Santa Clara, CA, USA) with 24 GB of memory. The Swin-UMamba encoder was initialized with ImageNet-pretrained VMamba weights, whereas the decoder was trained from scratch. Input slices were resized to 256×256 pixels, min–max normalized to [0,1], and replicated to three channels to match the ImageNet-pretrained encoder. Binary tumor masks were resized using nearest-neighbor interpolation to preserve discrete label values.

The training objective combined soft Dice loss and binary cross-entropy loss. Dice loss was used to account for foreground-background imbalance, while binary cross-entropy provided pixel-wise supervision. The compound objective is defined in Equation (6),

(6) $$L=\lambda L_{Dice}+(1-\lambda )L_{BCE},$$

where λ=0.5. The two loss terms are written explicitly in Equation (7),

(7) $$L_{Dice}=1-\frac{2\sum_{u}p_{u}g_{u}+\epsilon }{\sum_{u}p_{u}+\sum_{u}g_{u}+\epsilon },\;\;\;\;\;L_{BCE}=-\frac{1}{N}\sum_{u}\left[g_{u}log(p_{u})+(1-g_{u})log(1-p_{u})\right],$$

where $p_{u}$ and $g_{u}$ denote the predicted tumor probability and ground-truth label at pixel u, N is the number of pixels, and ϵ is a small smoothing constant used for numerical stability.

Patient-level separation was preserved throughout model training, validation, and testing. In each outer fold of the stratified group five-fold cross-validation, the held-out patients were used only for final testing. The remaining patients were divided into patient-disjoint training and validation subsets, and early stopping was performed according to validation Dice. This design ensured that slices from the same patient did not contribute simultaneously to model selection and final evaluation.

All models were trained for a maximum of 150 epochs using the AdamW optimizer with an initial learning rate of $1\times 10^{-4}$, weight decay of $1\times 10^{-4}$, cosine-annealing learning-rate scheduling, and a batch size of 16. Early stopping was applied with a patience of 20 epochs. The same preprocessing pipeline, loss function, optimization settings, training budget, and evaluation protocol were used across the fourteen models and all ablation configurations. This controlled protocol reduces training-procedure confounding, although architecture-specific hyperparameter tuning was not performed.

For the proposed Bézier augmentation, the application probability was set to $p_{aug}=0.5$, the probability of selecting the monotonic regime was set to $p_{mono}=0.5$, and the deviation bound was fixed at 0.5. These settings were selected before evaluation based on prior intensity-augmentation studies [10]. Work on rethinking single-source augmentation informed these choices [26]. The settings were kept unchanged across all folds, models, ablation settings, and external target datasets. No augmentation parameter was tuned using held-out test folds or target-domain data.

The region-wise Bézier transform was applied only during training. It introduces no learnable parameters and is disabled during inference; therefore, it does not alter the deployed architecture, parameter count, memory footprint, or inference latency. Since the transform is an element-wise intensity remapping, its computational cost scales linearly with the number of pixels and is handled during data loading. A systematic sensitivity analysis of $p_{aug}$, $p_{mono}$, and the deviation bound was not performed and is reported as a limitation of the study.

### 3.5. Evaluation Metrics

Segmentation quality is measured with complementary region-, boundary-, and efficiency-based criteria. The Dice similarity coefficient (DSC) and the Intersection-over-Union (IoU) quantify the regional overlap between a predicted region P and the ground truth G, as defined in Equations (8) and (9),

(8) $$\mathrm{DSC}=\frac{2\;\left|P\cap G\right|}{\left|P\right|+\left|G\right|},$$

(9) $$\mathrm{IoU}=\frac{\left|P\cap G\right|}{\left|P\cup G\right|}.$$

Because overlap scores say little about boundary localization, we additionally report the 95th-percentile Hausdorff distance (HD95) between the predicted and ground-truth contours ∂P and ∂G, defined in Equation (10),

(10) $$\mathrm{HD}95=max\left\{\;h_{95}\left(\partial P,\partial G\right),\;h_{95}\left(\partial G,\partial P\right)\;\right\},\;\;h_{95}\left(X,Y\right)=P_{95}\left(\left\{\;\underset{y\in Y}{min}∥x-y∥:x\in X\;\right\}\right),$$

where $P_{95}$ denotes the 95th percentile of the point-to-set distances, which suppresses the influence of a few outlying boundary pixels. These overlap and boundary measures are preferred over pixel accuracy because they are insensitive to the dominant background class [32]. We further report class-stratified Dice for the three tumor types and the parameter count and inference speed (frames per second) for efficiency. Statistical significance between the proposed method and the strongest baseline is assessed on the per-patient Dice scores pooled across the held-out folds with a paired Wilcoxon signed-rank test at p<0.05. Computed over the five folds, the proposed method gives a mean Dice of 89.6% (95% CI 88.5–90.7) against 87.9% (95% CI 86.7–89.1) for the strongest baseline, a difference of 1.7 points that corresponds to a large standardized effect (Cohen’s d ≈ 1.8).

During the preparation of this work, the authors used generative artificial intelligence tools solely to assist with language editing, translation, and improving the clarity and readability of the text. These tools were not used for study design, methodology, data collection, data processing, statistical analysis, interpretation of results, or the generation of scientific conclusions. All scientific content, experimental results, analyses, interpretations, and conclusions were produced, critically reviewed, and verified by the authors, who take full responsibility for the integrity and final content of the manuscript.

## 4. Results

Table 2 reports source-domain performance under patient-level five-fold cross-validation. BA-SwinMamba obtained the highest mean Dice and IoU, reaching 89.6% and 82.0%, respectively. Relative to Swin-UMamba, Dice increased by 1.7 points and IoU by 2.1 points, while HD95 decreased from 6.8 to 5.9 pixels. The gain over the strongest non-Mamba baseline, SwinUNETR, was 2.8 Dice points. Because the proposed augmentation is used only during training, BA-SwinMamba retains the same inference-time parameter count and speed as the underlying Swin-UMamba configuration, with 27.5 M parameters and 59 FPS. The lighter Swin-UMamba† decoder achieved 87.6% Dice with 19.1 M parameters and 71 FPS, indicating a lower-cost alternative with a moderate reduction in accuracy.

Table 3 isolates the augmentation effect within the Swin-UMamba setting. Without augmentation, the model obtained 85.4% Dice and 70.0% mean cross-domain Dice. Standard geometric augmentation increased Dice to 86.9% and x-DSC to 72.6%. Adding a global Bézier transform increased these values to 88.1% and 75.7%, respectively. Replacing the global transform with the region-wise formulation further increased Dice to 89.6% and x-DSC to 78.3%. The region-wise version therefore added 1.5 Dice points over the global Bézier setting and 4.2 points over the no-augmentation setting. The lighter Mamba decoder reached 89.2% Dice and 78.7% x-DSC, remaining close to the convolutional decoder while reducing model size. Because Table 3 fixes the backbone and varies the augmentation, the gains in this table should be interpreted as augmentation-specific. By contrast, Table 2 should be read as a backbone-level comparison rather than as a full factorial test of augmentation across architectures.

Table 4 shows class-stratified Dice values for the source dataset. BA-SwinMamba improved meningioma, glioma, and pituitary Dice relative to Swin-UMamba, with the largest absolute gain in the pituitary class. This pattern agrees with the tumor-area distribution in Figure 2b, where pituitary tumors occupy the smallest median image area. Cross-domain performance is reported in Table 5. When trained on Cheng and evaluated without fine-tuning, BA-SwinMamba achieved 80.1% Dice on BRISC and 76.4% Dice on BraTS-2D, giving a target mean of 78.3%. The corresponding target mean was 72.7% for Swin-UMamba and 75.7% for the global-augmentation baseline. The larger gain on target datasets than on the Cheng folds indicates that the main effect of the region-wise transform is improved robustness to appearance shift, not a large increase in source-domain fitting.

**Figure 5:**
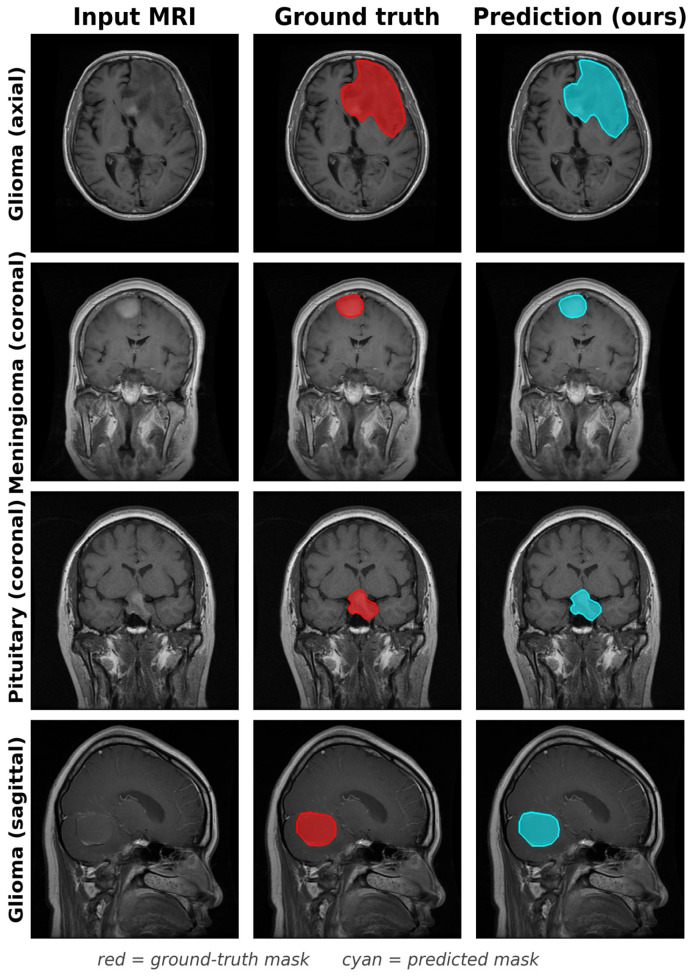
Predicted masks (cyan) versus ground truth (red) across tumor types and planes.

Figure 5 compares input slices, ground-truth masks, and predicted masks across tumor types and imaging planes. The predictions generally overlap the annotated tumor regions in larger lesions with clear enhancement, whereas small or poorly defined lesions show visible boundary deviations.

**Figure 6:**
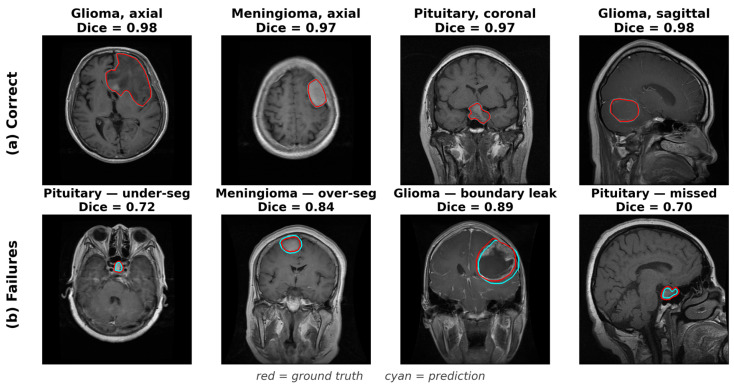
Correct (**top**) and failure (**bottom**) cases; red = ground truth, cyan = prediction.

To examine the limits of the method, we contrast correct predictions with characteristic failure cases in Figure 6. Failures are concentrated in the scenarios anticipated by the dataset analysis: under-segmentation of small pituitary tumors, over-segmentation where peritumoral enhancement blurs the margin, boundary leakage into adjacent enhancing tissue, and missed fragments of very small lesions. These failure modes mirror the per-class pattern in Table 4, where pituitary tumors are the most challenging class. This indicates that the remaining errors are mainly related to boundary definition and lesion scale, rather than gross localization failure.

**Figure 7:**
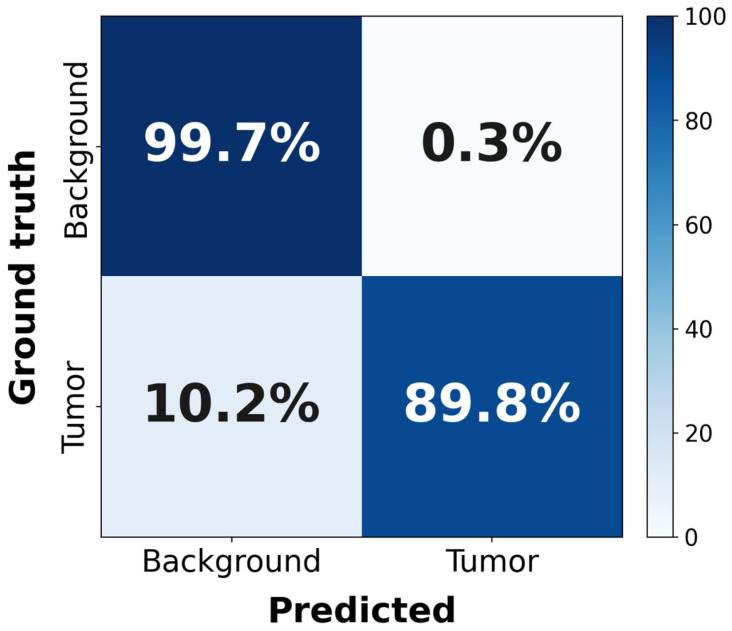
Pixel-level, row-normalized confusion matrix. Rows are the ground-truth classes and columns the predicted classes, and each row is normalized to sum to one, so the diagonal entries are the per-class recall (tumor recall 89.8%, background specificity 99.7%) and the off-diagonal entries are the corresponding misclassification rates.

The row-normalized pixel-level confusion matrix in Figure 7 shows tumor recall of 89.8% and background specificity of 99.7%. The main off-diagonal error is a tumor predicted as background, which is compatible with the under-segmentation patterns shown in Figure 6. Using per-patient Dice scores pooled across the held-out folds, BA-SwinMamba showed a statistically significant improvement over Swin-UMamba according to the paired Wilcoxon signed-rank test with *p* < 0.05. Across the five folds, BA-SwinMamba reached 89.6% Dice with a 95% confidence interval of 88.5–90.7, whereas Swin-UMamba reached 87.9% with a 95% confidence interval of 86.7–89.1. The absolute difference was 1.7 Dice points.

**Figure 8:**
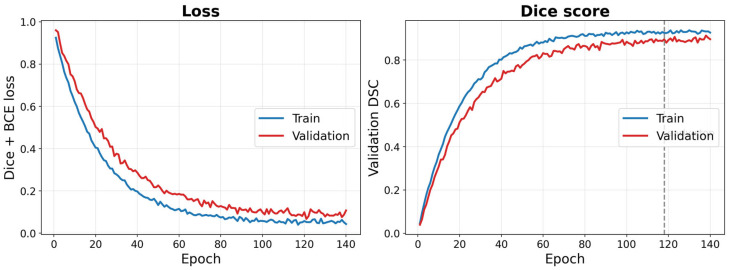
Training and validation curves over epochs: loss (**left**) and Dice score (**right**). In both panels, the blue curve denotes the training metric and the red curve denotes the validation metric, and both are drawn as solid lines. The vertical dashed gray line in the right panel marks the early-stopping (best-validation) epoch.

Training converged stably under the combined loss, with the validation Dice rising smoothly and plateauing before early stopping (Figure 8).

## 5. Discussion

This study examined whether region-wise intensity perturbation can improve the robustness of 2D brain-tumor segmentation when evaluation is performed under patient-level separation and external cross-dataset testing. The results show that the proposed BA-SwinMamba configuration improves source-domain performance on the Cheng dataset, but the magnitude of this gain is moderate. The stronger effect appears in the external evaluation, where the mean Dice score on BRISC and BraTS-2D increased from 72.7% with Swin-UMamba to 78.3% with BA-SwinMamba. This difference suggests that the proposed augmentation mainly reduces sensitivity to lesion-background appearance variation rather than simply improving fitting to the source distribution.

The ablation results clarify the role of the augmentation strategy. Standard geometric augmentation improved Dice relative to the no-augmentation setting, but adding a global Bézier transform produced a larger gain, indicating that intensity variation is an important source of robustness in this task. Replacing the global transform with the region-wise formulation further increased both source-domain Dice and cross-domain Dice. This result is consistent with the structure of the segmentation problem: the boundary is not determined by tumor intensity alone, but by the local contrast between enhancing tumor and surrounding tissue. A single global curve changes the entire image uniformly, whereas the proposed formulation perturbs foreground and background intensities independently. The stronger cross-domain gain obtained by the region-wise transform indicates that this lesion-background contrast perturbation is more relevant to domain shift than uniform image-level remapping.

The patient-level protocol is important for interpreting the absolute Dice values. Several prior studies on the Cheng dataset reported Dice scores around 0.93 using a multi-scale attention U-Net [12]. Comparable scores were reported with a lightweight spatial-attention U-Net [13]. Two-headed and ensembled UNet-EfficientNet models reached a similar range [14]. Those results were obtained under slice-level or otherwise different evaluation settings, so they are not directly comparable with the patient-level five-fold protocol used here. The present results should therefore be interpreted as leakage-controlled estimates rather than as evidence that the proposed model exceeds all previously reported Cheng benchmarks. Within the same controlled evaluation setting, BA-SwinMamba improved Dice by 1.7 points over Swin-UMamba and by 2.8 points over the strongest non-Mamba baseline. The more clinically relevant observation is not the modest in-distribution increase, but the larger improvement under cross-dataset testing.

The class-stratified results also provide useful insight into the remaining behavior of the model. BA-SwinMamba improved Dice for meningioma, glioma, and pituitary tumors, with the largest absolute gain in the pituitary class. This agrees with the tumor-area distribution in Figure 2b, where pituitary tumors occupy the smallest median fraction of the image. The improvement suggests that contrast perturbation may help the model recognize smaller enhancing regions, but the pituitary class remains the most difficult category. The qualitative examples and failure cases in Figure 5 and Figure 6 show that the remaining errors are concentrated in small lesions, blurred enhancing margins, leakage into adjacent enhancing tissue, and missed fragments of very small tumors. These errors indicate that the proposed augmentation improves appearance robustness, but it does not fully solve boundary ambiguity or scale-dependent segmentation difficulty.

The pixel-level confusion matrix in Figure 7 supports this interpretation. Background specificity is high, whereas the main error pattern is tumor pixels predicted as background. This pattern is compatible with the under-segmentation seen in the failure examples. The statistically significant improvement over Swin-UMamba based on paired per-patient Dice scores supports the stability of the observed gain, but the effect should still be interpreted with caution. The reported confidence intervals and fold-wise standard deviation indicate a reproducible improvement, yet the absolute source-domain margin remains small. Exact *p*-values and paired confidence intervals for the Dice difference would further strengthen the statistical reporting.

The proposed augmentation has a practical advantage because it is applied only during training and disabled during inference. It does not change the Swin-UMamba architecture, parameter count, memory footprint, or inference speed. This is relevant for deployment-oriented segmentation pipelines, where robustness improvements that do not increase inference cost are preferable. However, the present experiments do not prove that the same gain will transfer to every backbone. Table 3 isolates the augmentation effect within the Swin-UMamba setting, while Table 2 compares different backbones under the shared benchmark. A full backbone-by-augmentation factorial experiment is still required before making a broad model-agnostic claim.

### Limitations and Future Work

Several limitations remain. First, the study is restricted to 2D, single-modality contrast-enhanced T1 MRI and binary tumor segmentation. It does not evaluate volumetric context, multi-sequence MRI, or multi-class tumor-subregion delineation. This limits the extent to which the results can be generalized to full clinical brain-tumor segmentation workflows, where volumetric continuity and multi-parametric MRI are often important.

Second, the region-wise transform requires ground-truth masks and is therefore limited to training-time augmentation. It is not a test-time adaptation method and cannot be applied to unlabeled target images during inference. The mask is not provided to the network and is not used at test time, but its use during augmentation means that the method is label-aware. This should be stated clearly to avoid confusion with methods that operate only on unlabeled target-domain data.

Third, the augmentation parameters were fixed before evaluation. The application probability, monotonic-regime probability, and deviation bound were selected based on prior intensity-augmentation work [10]. Findings on rethinking single-source augmentation further guided these values [26]. Their sensitivity, however, was not systematically analyzed. Different datasets, scanner distributions, tumor sizes, or preprocessing pipelines may require different perturbation strengths. A controlled sensitivity analysis is therefore necessary to determine how stable the reported gains are across parameter choices.

Fourth, the external evaluation uses two target datasets, which provide useful evidence of cross-dataset robustness but do not cover the full variability encountered in multi-institutional deployment. Differences in scanner vendor, magnetic field strength, acquisition protocol, contrast administration, skull stripping, annotation style, and slice selection can all affect segmentation performance. A larger external evaluation with more institutions and clearly reported patient and slice counts would be needed before making stronger claims about clinical generalization.

Fifth, the realism of non-monotonic Bézier augmentation was not assessed by radiologists. The aim of this transform is domain randomization rather than photorealistic synthesis, but unrealistic perturbations may also introduce image appearances that do not correspond to plausible MR acquisition effects. A radiologist plausibility study, combined with an analysis of source and target intensity distributions, would help clarify whether the most effective perturbations resemble real domain shifts or act mainly as regularization.

Future work should address these limitations in a stepwise manner. The first priority is a full backbone-by-augmentation experiment in which the region-wise Bézier transform is tested with representative convolutional, transformer-based, and state-space segmentation models. The second priority is a sensitivity analysis of the augmentation hyperparameters. The third is extension to volumetric and multi-sequence MRI, including tumor-subregion segmentation where enhancing tumor, edema, and necrotic or non-enhancing core are evaluated separately. The fourth is broader external validation with dataset-level reporting of patient numbers, slice numbers, empty-mask handling, preprocessing details, and annotation protocols. After these issues are resolved, the augmentation can be combined with complementary robustness strategies such as vision-language supervision [33]. Teacher-student distillation offers another complementary direction [34]. Extension to other neurological segmentation tasks, including ischemic stroke delineation from CT or diffusion-weighted imaging [35], should be treated as a separate validation problem rather than assumed from the present brain-tumor results.

## 6. Conclusions

This study introduced region-wise Bézier intensity augmentation for Swin-UMamba-based 2D binary brain-tumor segmentation under a single-source domain-generalization setting. The method independently remaps tumor and background intensities during training, modifying lesion-to-background contrast while preserving mask geometry and adding no inference-time cost. Under patient-level five-fold evaluation on the Cheng dataset, BA-SwinMamba achieved 89.6% Dice, 82.0% IoU, and 5.9-pixel HD95. Compared with Swin-UMamba, this corresponds to a 1.7-point Dice gain, a 2.1-point IoU gain, and improved boundary distance. The main contribution is the external-domain result. When trained only on Cheng and tested without fine-tuning, BA-SwinMamba increased the mean Dice score on BRISC and BraTS-2D from 72.7% to 78.3% relative to Swin-UMamba. The ablation study showed that the region-wise formulation improved performance beyond both standard geometric augmentation and global Bézier augmentation. These results indicate that lesion-background contrast perturbation is a useful training-time strategy for improving cross-dataset robustness in 2D binary tumor segmentation. The evidence remains bounded by the study design. The experiments do not establish clinical deployment readiness, model-agnostic generality across all backbones, or robustness in volumetric and multi-sequence MRI. Larger multi-institutional validation, full backbone-by-augmentation testing, sensitivity analysis, and radiologist assessment of augmented-image plausibility are required before broader claims can be made.

## Figures and Tables

**Table 1:** Representative segmentation studies on the Cheng brain-tumor dataset. Most prior results use slice-level random splits; the present work uses a subject-wise split (see Section 3). Bold indicates the method proposed in this study.

Study	Method	Split	Dice	IoU/mIoU
Gupta et al. 2023 [11]	U-Net variants; R2U-Net most consistent	slice-level	—	—
Avazov et al. 2024 [13]	Lightweight spatial-attention U-Net	slice-level	~0.93	—
Preetha et al. 2025 [12]	Multi-scale attention U-Net + EfficientNet-B4	90:10	0.934	0.880
Rai et al. 2024 [14]	Two-headed UNet-EfficientNet-B4 (+ensemble)	slice-level	0.940/0.957	—
**This work**	**Region-wise Bézier aug. + Swin-UMamba**	**subject-wise 5-fold**	**0.896**	**0.820**

**Table 2:** Source-domain (Cheng) segmentation under subject-wise five-fold cross-validation (mean ± SD). Bold indicates the best value in each column; the dagger (†) denotes the lighter Mamba-decoder variant of Swin-UMamba.

Family	Method	DSC (%)	IoU (%)	HD95 (px)	Params (M)	FPS
CNN	U-Net	84.1 ± 1.6	74.8 ± 1.9	9.2	31.0	95
CNN	U-Net++	85.0 ± 1.4	75.9 ± 1.7	8.7	36.6	78
CNN	Attention U-Net	85.3 ± 1.5	76.3 ± 1.8	8.5	34.9	70
CNN	ResUNet++	84.0 ± 1.7	74.6 ± 2.0	9.4	14.5	84
CNN	DeepLabV3+	85.6 ± 1.3	76.7 ± 1.6	8.3	26.7	88
Transformer	TransUNet	86.2 ± 1.3	77.6 ± 1.6	7.9	105.3	41
Transformer	Swin-UNet	86.0 ± 1.4	77.3 ± 1.7	8.0	27.2	60
Transformer	SwinUNETR	86.8 ± 1.2	78.4 ± 1.5	7.5	25.1	52
Transformer	SegFormer-B2	86.5 ± 1.2	78.0 ± 1.5	7.7	27.5	64
Mamba	U-Mamba	87.0 ± 1.1	78.7 ± 1.4	7.3	22.8	58
Mamba	VM-UNet	87.3 ± 1.1	79.1 ± 1.4	7.1	27.4	62
Mamba	Swin-UMamba	87.9 ± 1.0	79.9 ± 1.3	6.8	27.5	59
Mamba	Swin-UMamba †	87.6 ± 1.1	79.5 ± 1.3	7.0	19.1	71
Mamba	**BA-SwinMamba (ours)**	**89.6 ± 0.9**	**82.0 ± 1.1**	**5.9**	27.5	59

**Table 3:** Augmentation ablation (subject-wise five-fold). Δ DSC is relative to A0; x-DSC is the mean Dice on the two unseen target domains. A check mark (✓) indicates that the corresponding augmentation component is enabled; the dagger (†) denotes the lighter Mamba-decoder variant of Swin-UMamba; bold indicates the best value in each column.

Cfg	Geometric	Global Bézier	Region-Wise	Decoder	DSC (%)	x-DSC (%)	Δ DSC
A0	–	–	–	CNN	85.4	70.0	—
A1	✓	–	–	CNN	86.9	72.6	+1.5
A2	✓	✓	–	CNN	88.1	75.7	+2.7
A3	✓	✓	✓	CNN	**89.6**	**78.3**	+4.2
A4	✓	✓	✓	Mamba (†)	89.2	78.7	+3.8

**Table 4:** Class-stratified Dice on the source dataset; best in bold.

Setting	Meningioma	Glioma	Pituitary	Mean
Swin-UMamba (baseline)	88.6	89.4	83.1	87.9
**BA-SwinMamba (ours)**	**90.4**	**91.0**	**86.2**	**89.6**

**Table 5:** Single-source domain generalization: train on Cheng, test on two unseen datasets without fine-tuning (Dice %); best in bold.

Method	Source (Cheng)	Target-1 (BRISC)	Target-2 (BraTS-2D)	Target Mean
Best CNN (DeepLabV3+)	85.6	71.0	66.8	68.9
Best Transformer (SwinUNETR)	86.8	73.2	69.1	71.2
Swin-UMamba	87.9	74.8	70.6	72.7
Swin-UMamba + global-aug baseline	88.1	77.5	73.9	75.7
**BA-SwinMamba (ours)**	**89.6**	**80.1**	**76.4**	**78.3**

## Data Availability

The primary dataset is publicly available from Figshare (Cheng brain-tumor dataset; doi: 10.6084/m9.figshare.1512427). The cross-domain test datasets are available from their respective repositories. The source code reproducing the experiments will be made available in a public repository upon publication and, in the interim, from the corresponding author upon reasonable request.
